# Brevetoxin versus Brevenal Modulation of Human Nav1 Channels

**DOI:** 10.3390/md21070396

**Published:** 2023-07-07

**Authors:** Rocio K. Finol-Urdaneta, Boris S. Zhorov, Daniel G. Baden, David J. Adams

**Affiliations:** 1 Illawarra Health & Medical Research Institute (IHMRI), Faculty of Science, Medicine and Health, University of Wollongong, Wollongong, NSW 2522, Australia; 2 Department of Biochemistry and Biomedical Sciences, McMaster University, Hamilton, ON L8S 4K1, Canada; zhorov@mcmaster.ca; 3 I.M. Sechenov Institute of Evolutionary Physiology and Biochemistry, Russian Academy of Sciences, 194223 Saint Petersburg, Russia; 4 Shemyakin-Ovchinnikov Institute of Bioorganic Chemistry, Russian Academy of Sciences, 117997 Moscow, Russia; 5 Center for Marine Science, University of North Carolina Wilmington MARBIONC, Wilmington, NC 28409, USA; biotechbaden@gmail.com

**Keywords:** brevenal, brevetoxins, Nav, ladder-frame polyethers, PbTx-3, PbTx-2, voltage-gated sodium channels

## Abstract

Brevetoxins (PbTx) and brevenal are marine ladder-frame polyethers. PbTx binds to and activates voltage-gated sodium (Nav) channels in native tissues, whereas brevenal antagonizes these actions. However, the effects of PbTx and brevenal on recombinant Nav channel function have not been systematically analyzed. In this study, the PbTx-3 and brevenal modulation of tissue-representative Nav channel subtypes Nav1.2, Nav1.4, Nav1.5, and Nav1.7 were examined using automated patch-clamp. While PbTx-3 and brevenal elicit concentration-dependent and subtype-specific modulatory effects, PbTx-3 is >1000-fold more potent than brevenal. Consistent with effects observed in native tissues, Nav1.2 and Nav1.4 channels were PbTx-3- and brevenal-sensitive, whereas Nav1.5 and Nav1.7 appeared resistant. Interestingly, the incorporation of brevenal in the intracellular solution caused Nav channels to become less sensitive to PbTx-3 actions. Furthermore, we generated a computational model of PbTx-2 bound to the lipid-exposed side of the interface between domains I and IV of Nav1.2. Our results are consistent with competitive antagonism between brevetoxins and brevenal, setting a basis for future mutational analyses of Nav channels’ interaction with brevetoxins and brevenal. Our findings provide valuable insights into the functional modulation of Nav channels by brevetoxins and brevenal, which may have implications for the development of new Nav channel modulators with potential therapeutic applications.

## 1. Introduction

**Figure 1:**
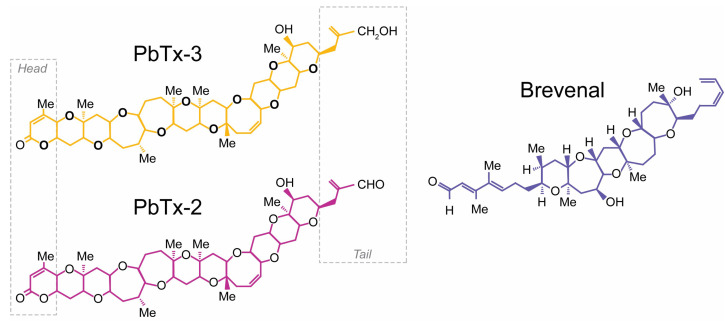
Structure of brevetoxins PbTx-3 (gold), PbTx-2 (magenta), and brevenal (blue). The six-membered ring lactone (Head) and side chain (Tail) of the brevetoxins are shown within gray boxes.

Ladder-frame polyethers (LFP) constitute a well-studied family of marine compounds encompassing the brevetoxins (PbTx-#, Figure 1) and ciguatoxins produced by *Karenia* and *Gambierdiscus* dinoflagellates, respectively [1,2,3]. Brevetoxins were first discovered during harmful algal blooms in the early 1970s, causing severe respiratory dysfunction in beach-goers [4,5]. Over the next two decades, twelve different natural brevetoxins were isolated and characterized from *Karenia brevis* cultures [6,7]. Inhaling these toxins can cause bronchoconstriction, decreased mucociliary clearance, and decreases in the rate of whole-lung clearance in animal models of cystic fibrosis [8].

All brevetoxins described to date interact with site 5 of voltage-gated sodium (Nav) channels and act as channel activators [9,10,11,12]. Natural brevetoxins induce four distinct changes to sodium channel activity: (1) a shift in activation to more negative potentials; (2) longer mean open times, (3) inhibition of fast inactivation, and (4) a set of normal and partially inhibited channel sub-conductance states [13]. Derivatized brevetoxins have been coined “molecular measuring tapes” due to their inherent structural rigidity. These derivatives offer valuable insights into the regions involved in key molecular events of Nav channel functional traits such as voltage dependence, inactivation, and the appearance of sub-conductance states.

Studies of the relationship between the structure of PbTx-2 and its ability to bind to and activate neuronal Nav channels revealed key structural features essential to brevetoxin activities [14,15,16,17]. Composed of a “head” region (ring A), a “tail region” (composed of the terminal four rings), and a “spacer region” (composed of rings B-G between the head and tail), brevetoxin modified in the spacer region (truncated PbTx-2) reduces potency, whilst the presence of modified functional groups on the A-ring increases the breadth of observed sub-conductance states, and changes in the terminal side chain tend to increase affinity, which affect their potency [18]. Furthermore, different Nav channel actions could be ascribed to specific molecular derivatives [18]. One derivative, 2,3-dihydro PbTx-3 A-ring diol, specifically induced sub-conductance states. Most derivatives knocked out longer mean open time and inhibition of inactivation. One bulky side chain derivative acted as a Na^+^ influx inhibitor. In addition, the binding constants for brevetoxin derivatives most characteristically exhibit reduced affinity for site 5 on the α-subunit of sodium channels. Any brevetoxin derivative that is still bound to site 5 exhibited some ability to shift activation to more negative potentials [18].

In 2004, a novel brevenal polyether structure was discovered amongst the compounds isolated from *K. brevis* cultures. This compound (Figure 1) [19] acts as a sodium channel blocker and antagonizes the effects of brevetoxin and ciguatoxin administration [20]. It is important to note that brevenal is shorter than either class of brevetoxins and that it does not possess a lactone moiety in the A-Ring, nor does it possess an alpha, beta-unsaturated aldehyde or reduced aldehyde. When administered alone, brevenal did not cause bronchoconstriction but increased mucociliary clearance and ciliary beat frequency. It also increased the rate of whole-lung clearance in animal models of cystic fibrosis. Pretreatment with brevenal before exposure to brevetoxins prevented toxin effects. Co-administration of brevenal with brevetoxin resulted in a reduction in toxin effects, and post-toxin treatment with brevenal antagonized toxin effects in both animal models [21] and binding assays [22]. Brevenal reduced brevetoxin binding in radioligand assays [22,23], and decreased the immunological effects in plaque-forming cells and DNA damage caused by toxins [24,25,26]. Radioligand cross-over experiments showed that brevenal could reduce tritium-labeled brevetoxin binding, but brevetoxin could not inhibit tritiated brevenol binding [22]. Thus, a mechanism of competition or overlap between brevetoxin and brevenal binding sites was postulated. Moreover, brevenal’s ability to displace brevetoxin from its binding site suggests that the brevenal binding locus is part of the essential binding site for brevetoxin [22].

While much research has focused on the binding interaction of brevetoxin(s) and brevenal LFPs in native tissues and primary cells, complementary functional studies in recombinant systems have been lacking. The heterologous expression of channel subtypes in cells provides a model system to explore toxin and antitoxin subtype specificity. By employing binding assays coupled with alanine scanning mutagenesis (ASM) of the Nav1.2 channel, recent research demonstrated that [42-^3^H]-PbTx-3 interacts with transmembrane helices IS6, IVS5, and IVS6 in multiple distributed interactions [12]. Despite these findings, experimental 3D structures of Nav channels interacting with brevetoxins and/or brevenal have remained unavailable.

Our current knowledge of how brevetoxins affect voltage-gated sodium channels is primarily based on binding assays and excitability studies conducted on native tissues from various species such as crayfish, squid, and rodents, along with single-channel recordings of native sodium channels. To expand on this knowledge, our study compared the effects of PbTx-3 and brevenal on macroscopic currents mediated by a variety of recombinant Nav channel subtypes expressed in mammalian cells. We utilized automated patch-clamp electrophysiology to investigate the functional impact of PbTx-3 and brevenal on human Nav channels and employed computational modeling to validate the binding of brevetoxin and brevenal to Nav channel pharmacological site 5. The results of our study provide valuable insights into the functional modulation of Nav channels by polyether ladder compounds, which may have implications for the development of new Nav channel modulators with potential therapeutic applications.

## 2. Results

Functional modulation of recombinant human voltage-gated sodium (Nav) channels exposed to marine PbTx-3 and brevenal was assessed by automated patch-clamp electrophysiology. Neuronal Nav1.2 (CNS) and Nav1.7 (PNS), in addition to muscle Nav1.4 (skeletal) and Nav1.5 (cardiac) sodium channels, were patch-clamped in mammalian cells. Whole-cell currents mediated by human Nav1.4 and Nav1.7 overexpressed in CHO-K1 cells, as well as human Nav1.2 and Nav1.5 overexpressed in HEK293 cells, were recorded at room temperature. Currents were elicited by 25 ms test pulses to −20 mV from a holding potential (Vh) of −120 mV, delivered for 5 min at 0.1 Hz, after each full-bath solution exchange to allow current amplitude and kinetics to reach a steady state.

**Figure 2:**
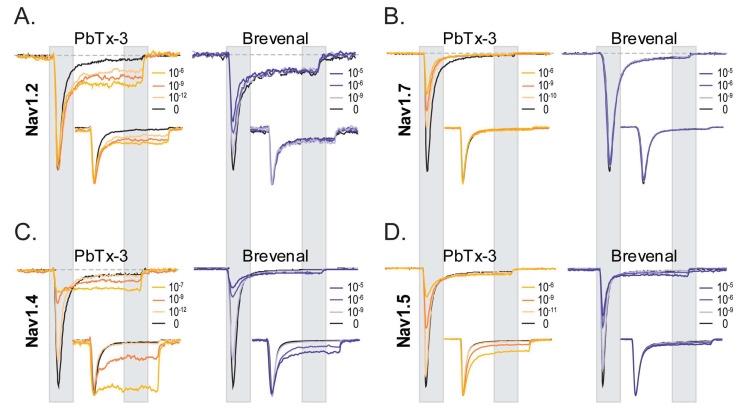
PbTx-3 and Brevenal differentially modulate Na^+^ currents mediated by human neuronal and muscle Nav channels. Representative whole-cell current traces mediated by Nav1.2 (**A**), Nav1.7 (**B**), Nav1.4 (**C**), and Nav1.5 (**D**) channels recorded by automated patch-clamp (APC) exposed to increasing concentrations of PbTx-3 (gold, 10^−12^–10^−6^ M) and brevenal (blue, 10^−9^–10^−5^ M); control is shown in black. Insets display scaled traces to highlight current kinetics. Test pulse: 25 ms, −20 mV, Vh −120 mV, 0.1 Hz. Peak and sustained currents are indicated by the gray shading.

Figure 2 displays representative current traces of neuronal (Figure 2A,B) and muscle Nav channels (Figure 2C,D) in control (black) and upon extracellular application of brevetoxin 3 (PbTx-3, gold) and brevenal (blue), highlighting the key features of PbTx-3- and brevenal-induced modifications of human Nav-mediated currents.

### 2.1. Distinct Functional Effects of PbTx-3 and Brevenal over Human Nav Channels

A salient observation from the shown experiments is the differential effects of PbTx-3 and brevenal on different isoforms of Nav channels. Under the experimental conditions used, both PbTx-3 and brevenal exerted subtype-specific modulatory effects on currents mediated by recombinant human neuronal and muscle Nav channel isoform in a concentration-dependent manner (Figure 2). In the presence of PbTx-3, an enhancement of the late component (I_late_ = average current between 85% and 95% of the pulse duration) of Nav1.2 currents elicited by a depolarizing step to −20 mV without apparent changes to the peak current amplitude (I_peak_ = maximal current between 5 and 15% of the pulse duration) (Figure 2A, left). Brevenal, in turn, modestly inhibited Nav1.2’s I_peak_, without exerting apparent changes to I_late_ when compared to the control condition (Figure 2A, Table 1). In contrast, while PbTx-3 inhibited Nav1.7 I_peak_ and failed to enhance its I_late_ component (Figure 2B, left), brevenal did not modulate Nav1.7 currents (up to 30 μM) (Figure 2B, Table 1). PbTx-3 and brevenal inhibited Nav1.4 peak currents while significantly enhancing the late current component (Figure 2C, Table 1). At Nav1.5, inhibition of I_peak_ at higher PbTx-3 and brevenal concentrations was evidenced, but although modest enhancement of I_late_ was observed, this did not reach statistical significance when compared to the control (Figure 2D, Table 1). These observations feature distinct functional effects of polyether brevetoxin compounds’ modification of recombinant neuronal and muscle Nav channels.

Current traces were scaled to maximal I_peak_ and are presented as insets in Figure 2 to underline the effects (or lack thereof) of PbTx-3 and brevenal on the current kinetics of the four Nav isoforms studied. Inactivation time constants (τ_inact_) in control and the presence of PbTx-3 and brevenal were estimated by exponential fits (see Methods) to the current decay between 15% and 85% of the depolarizing pulse. Single exponential fits adequately described τ_inact_ for the neuronal and the skeletal muscle Nav isoforms, whereas a double exponential fit was required to approximate open channel inactivation of the cardiac Nav current. Consistent with the enhancement of I_late_ observed for Nav1.2, Nav1.4, and Nav1.5 in the presence of PbTx-3, significant concentration-dependent increases in the Y_inf_ asymptote were detected (Tables S1–S3). These were mirrored by brevenal exposure in Nav1.2- and Nav1.4-mediated currents. Nevertheless, the estimated τ_inact_s appeared insensitive to PbTx-3 and brevenal modification, with the notable exception of Nav1.4, for which high concentrations of PbTx-3 and brevenal caused a measurable slowing of the open channel inactivation time constant (Table S2).

### 2.2. Distinct Potency of PbTx-3 and Brevenal over Human Nav Channels

**Figure 3:**
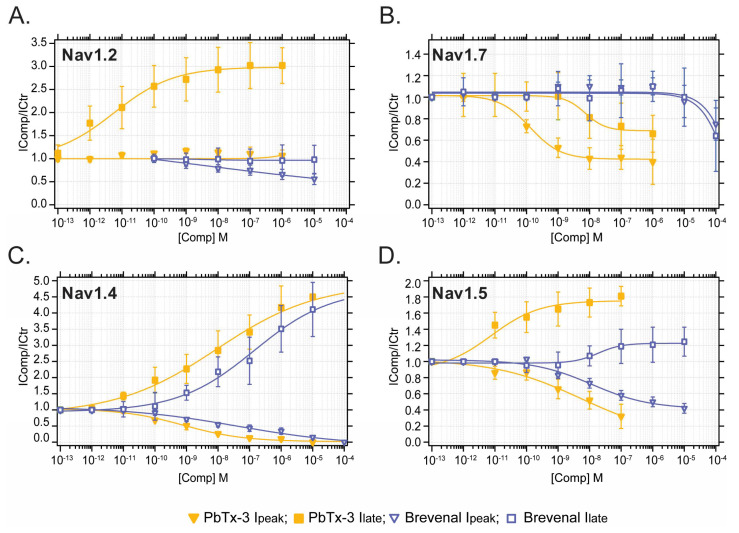
Differential potency of PbTx-3 and brevenal in the modulation of human Nav channel-mediated currents. (**A**) Nav1.2, (**B**) Nav1.7, (**C**) Nav1.4 and (**D**) Nav1.5. ▼ PbTx-3 I_peak_; ■ PbTx-3 I_late_; ▽ Brevenal I_peak_; ☐ Brevenal I_late_. Data represent mean ± SEM, *n* = 5 for all determinations.

Concentration–response curves (CRC) were built for the modification of I_peak_ (squares) and I_late_ (downward triangles) mediated by neuronal and muscle Nav currents (Figure 3) emphasizing subtype-specific potencies by PbTx-3 and brevenal.

In general, PbTx-3 was 1–3 orders of magnitude more potent than brevenal at modulating Nav currents with CRC-derived half inhibitory or effective concentrations (EC_50_) of 10^−8^–10^−12^ M and 10^−5^–10^−9^ M (pI/EC_50_ 7–11 and 5–8), respectively (Figure 3, Table 1). A comparison of PbTx-3 and brevenal potency for the enhancement of I_late_ and inhibition of I_peak_ for all Nav tested is provided in Figure S1. Specifically, PbTx-3 exposure resulted in a 2–4-fold enhancement of late currents mediated by Nav1.2, Nav1.4, and Nav1.5 with EC_50_s in picomolar to nanomolar values (Figure 3A,C,D). In contrast, PbTx-3 incompletely inhibited Nav1.7’s I_late_ (IC_50_ 34 nM, maximal block 60%) (Figure 3B, Table 1). Furthermore, PbTx-3 potently inhibited I_peak_ in Nav1.4, Nav1.5, and Nav1.7. However, complete blocking was observed only for Nav1.4 within the attainable concentration range of PbTx-3 (Figure 2 and Figure 3, Table 1).

Brevenal modestly modulated peak Nav currents with the following order of inhibitory potency: Nav1.4 > Nav1.2 > Nav1.5 >>> Nav1.7 (Figure 3, Table 1). Interestingly, brevenal (FC^Max^ = 4.1) was as effective as PbTx-3 (FC^Max^ = 4.5) at enhancing Nav1.4-mediated I_late_ but had ~25-fold lower potency (Brevenal EC_50_ 100 nM vs. PbTx-3 EC_50_ 4 nM) and failed to modulate I_late_ in any other Nav isoforms at the concentrations tested (Figure 3, Table 1). These observations highlight the apparent highest potency of PbTx-3 and brevenal for the mammalian skeletal muscle Nav1.4, followed by CNS’s Nav1.2, and lower potency for cardiac Nav1.5 and peripheral Nav1.7 channels.

### 2.3. Voltage Dependence of PbTx-3- and Brevenal-Modified Nav Channels

The voltage dependence of recombinant Nav channel-mediated currents exposed to PbTx-3 and brevenal was evaluated using current–voltage (I-V) relationships built for I_peak_ and I_late_ elicited by a standard stimulation protocol (25 ms, −100 to +60 mV, Vh −120 mV, 0.1 Hz).

The effects of extracellular exposure to PbTx-3 (PbTx-3_o_ = 1 μM) and brevenal (Brevenalo = 10 μM) on the activation of Nav channels in neuronal and muscle cells were examined under normal intracellular ionic conditions. These experiments revealed subtype-specific effects of PbTx-3 and brevenal. To investigate the possibility of overlapping binding of these compounds within Nav channels, Nav whole-cell currents were recorded using an intracellular solution supplemented with brevenal (Brevenali = 1 μM). After equilibration, extracellular PbTx-3o (1 μM) was applied and its modulatory effects over the current kinetics and voltage dependence of activation were compared.

**Figure 4:**
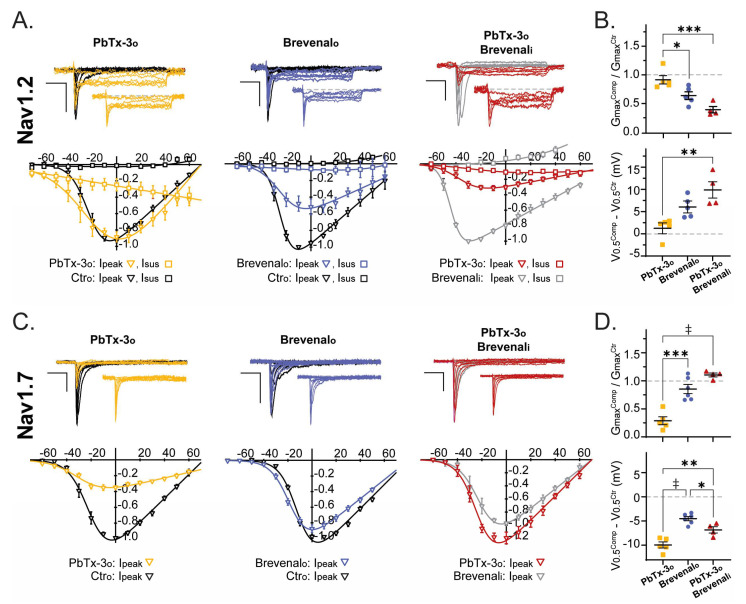
PbTx-3 and brevenal differentially modulate the kinetics of neuronal Nav-mediated currents. (**A**,**C**) Representative whole-cell current traces (top) and I-V plots (bottom) in response to a standard stimulation protocol (25 ms, −100 to +60 mV, Vh −120 mV, 0.1 Hz) from recombinant Nav1.2 and Nav1.7, respectively, recorded in different experimental conditions: *i*. control intracellular and extracellular absence of toxin (Ctro: black); *ii*. control intracellular and extracellular PbTx-3o (gold, ▼ 1 nM, ▽ 1 μM), or Brevenalo (blue, 10 μM); *iii*. brevenal in intracellular without toxin in extracellular (Brevenali: gray); *iv*. brevenal in intracellular and PbTx-3 in extracellular (PbTx-3o/Brevenali: magenta). Nav1.4: Brevenali 0.1 μM, PbTx-3o 1 nM; Nav1.5: Brevenali 1 μM, PbTx-3o 1 μM. Scale bars: 1 nA, 5 ms. (**B**,**D**) Scatter plots of the relative maximal macroscopic conductance (Gmax^Tox^/Gmax^Ctr^, top), and shift in the voltage dependence of activation (V0.5^Tox^ − V0.5^Ctr^ in mV, bottom) of peak currents mediated by Nav1.2 (**B**) and Nav1.7 (**D**) in the presence of PbTx-3o (■), Brevenalo (⬤), and PbTx-3o/Brevenali (▲). One-way ANOVA with Tukey’s multiple comparisons test, * *p* < 0.05; ** *p* ≤ 0.01; *** *p* ≤ 0.001; ‡ *p* < 0.0001.

**Figure 5:**
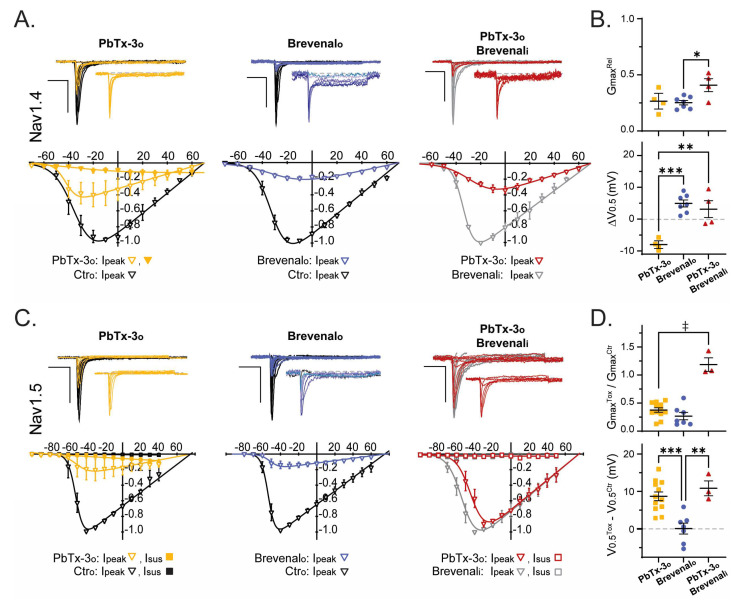
PbTx-3 and brevenal differentially modulate the kinetics of muscle Nav-mediated currents. (**A**,**C**) Representative whole-cell current traces (top) and I-V plots (bottom) in response to a standard stimulation protocol (25 ms, −100 to +60 mV, Vh −120 mV, 0.1 Hz) from recombinant Nav1.4 and Nav1.5, respectively, recorded in different experimental conditions: *i*. control intracellular and extracellular absence of toxin (Ctro: black); *ii*. control intracellular and extracellular PbTx-3o (gold, ▼ 1 nM, ▽ 1 μM) or Brevenalo (blue, 10 μM); *iii*. Brevenal in intracellular without toxin in extracellular (Brevenali: gray); *iv*. brevenal in intracellular and PbTx-3 in extracellular (PbTx-3o/Brevenali: magenta). Nav1.4: Brevenali 0.1 μM, PbTx-3o 1 nM; Nav1.5: Brevenali 1 μM, PbTx-3o 1 μM. Scale bars: 1 nA, 5 ms. (**B**,**D**) Scatter plots of the relative maximal macroscopic conductance (Gmax^Tox^/Gmax^Ctr^, top), and shift in the voltage dependence of activation (V0.5^Tox^ − V0.5^Ctr^ in mV, bottom) of peak currents mediated by Nav1.4 (**B**) and Nav1.5 (**D**) in the presence of PbTx-3o (■), Brevenalo (⬤), and PbTx-3o/Brevenali (▲). One-way ANOVA with Tukey’s multiple comparisons test, * *p* < 0.05; ** *p* ≤ 0.01; *** *p* ≤ 0.001; ‡ *p* < 0.0001.

Figure 4 and Figure 5 display representative current traces mediated by human recombinant Nav channels in control and upon exposure to PbTx-3 and brevenal. I-V plots from the normalized peak and late (when present) currents amplitudes (I_peak_/I_peak_^max^ and I_late_/I_peak_^max^) were fitted to a Boltzmann function rendering the half-maximal activation potential (V_0.5_) and normalized macroscopic conductance (Gmax) parameters (see Section 4). The changes in V_0.5_ (ΔV_0.5_ = V_0.5_^Comp^ − V_0.5_^Ctr^) and relative Gmax (Gmax^Rel^ = Gmax^Comp^/Gmax^Ctr^) observed in the presence of PbTx-3 and brevenal and control conditions were then calculated and compared for all assayed conditions.

Extracellular exposure to PbTx-3 (1 μM) did not significantly modify the voltage dependence (ΔV_0.5_ = 1.5 ± 1.0 mV, *n* = 5) or the macroscopic conductance (Gmax^Rel^ = 0.9 ± 0.1, *n* = 5) of Nav1.2-mediated currents (Figure 4A,B); however, broadening of the peak I-V curve became evident (Figure 4A, left). Evaluation of the Nav1.2 late current component upon exposure to PbTx-3_o_ revealed a monotonically increasing I_late_ with progressive depolarization that reached up to ~40% of I_peak_ at potentials more positive than 40 mV (☐). This non-inactivating current likely underlies the apparent broadening of the peak I-V curve that was most evident in the shoulders of the potential range (Figure 4A, left). In contrast, exposure to brevenal_o_ (10 μM) leads to a ~40% decrease in Nav1.2 Gmax (Gmax^Rel^ = 0.6 ± 0.1, *n =* 5) and a small depolarizing shift in V_0.5_ compared to control (ΔV_0.5_ = 6.1 ± 1.0 mV, *n* = 5) (Figure 4B). Intracellular dialysis with brevenal (1 μM, Figure 4A, right) lead to a ~20 mV leftward shift in Nav1.2 V_0.5_ compared to control extracellular/intracellular conditions (Ctro:Brevenali = −43.9 ± 2.3 mV, *n* = 4, vs. Ctro:Ctri = −22.6 ± 1.9 mV, *n* = 5; *t*-test *p* = 0.0002). Moreover, in Brevenal_i_-occupied Nav1.2 channels, the extracellular application of PbTx-3 (1 μM) elicited a smaller monotonic I_late_ (than that observed in PbTx-3o/Ctri), together with significant changes to G_max_ and V_0.5_ (Figure 4B).

Interestingly for Nav1.7, PbTx-3_o_ exposure led to a ~70% decrease in maximal conductance (Gmax^Rel^ = 0.3 ± 0.1, *n =* 5) and a −10.0 ± 0.6 mV (*n =* 5) shift in the voltage dependence of activation. In comparison, the Brevenalo-induced decrease in Nav1.7’s Gmax was ~10% (Gmax^Rel^ = 0.9 ± 0.1, *n =* 5; unpaired *t*-test *p* = 0.0006) with a shift in V_0.5_ of −4.5 ± 0.4 mV (*n* = 6; unpaired *t*-test *p* < 0.0001) (Figure 4C,D). The voltage dependence of activation of Brevenali-occupied Nav1.7 channels (V_0.5_ = −16.6 ± 1.7 mV, *n* = 4) was indistinguishable from that observed in the control intracellular solution (V_0.5_ = −20.0 ± 2.4 mV, *n =* 5; unpaired *t*-test *p* = 0.3096). Whilst the extracellular application of PbTx-3 (1 μM) was ineffective at decreasing Gmax (Gmax^Rel^ = 1.1 ± 0.03, *n* = 4), a leftward shift in activation ΔV_0.5_ of 6.8 ± 0.7 mV (*n* = 4) was detected, which was significantly smaller than that observed in the absence of Brevenali (PbTx-3o:Ctri, one-way ANOVA *p* = 0.0078) (Figure 4C,D).

#### Muscle Navs

For Nav1.4-mediated currents, both PbTx-3o (1 nM) and Brevenalo (1 μM) led to ~70% inhibition of Gmax (Gmax^Rel^: 0.3 ± 0.05, *n* = 4 and 0.3 ± 0.02, *n* = 7, respectively). However, PbTx-3o caused a 9.2 ± 1.5 mV (*n* = 4) hyperpolarizing shift in V_0.5_, whereas the shift was in the depolarizing direction and 4.9 ± 1.1 mV (*n* = 7) in magnitude in Brevenalo (unpaired *t*-test *p* < 0.0001) (Figure 5E,F). Interestingly, exposure to a Nav1.4-saturating concentration of PbTx-3o (1 μM) completely inhibited I_peak_ effectively isolating a monotonic I_late_ component (▼) in the skeletal muscle Nav channel. The presence of brevenal (1 μM) in the intracellular solution was used to record Nav1.4 currents’ right-shifted activation by ~5 mV (V_0.5_: Ctro:Ctri −31.8 ± 0.5 mV, *n* = 7, vs. Ctro:Brevenali −26.4 ± 2.8 mV, *n* = 4; unpaired *t-test p* = 0.0324). Brevenali-occupied Nav1.4 channels were inhibited by PbTx-3 (Gmax^Rel^ = 0.4 ± 0.1, *n* = 4); however, it did not significantly alter activation V_0.5_ (ΔV_0.5_ = 3.1 ± 2.6 mV, *n* = 4) (Figure 5B).

The maximal conductance of cardiac Nav1.5 channels was inhibited by PbTx-3 (1 μM) and brevenal (10 μM) with relative Gmax of 0.4 ± 0.04 (*n* = 12) and 0.3 ± 0.1 (*n* = 7), respectively. Nav1.5 I_peak_ inhibition by PbTx-3_o_ (1 μM) was accompanied by a depolarizing 8.7 ± 1.2 mV (*n* = 12) shift in V_0.5_, whereas Brevenal_o_ (10 μM) did not alter the voltage dependence of activation of the cardiac Nav channel (ΔV_0.5_ = 0.1 ± 1.4 mV, *n* = 7). In Brevenal_i_-occupied cardiac channels, PbTx-3 was ineffective at inhibiting Gmax (PbTx-3_o_:Brevenal_i_ Gmax^Rel^ = 1.2 ± 0.1, *n* = 3), but similar to PbTx-3_o_:Ctri, a depolarizing shift in ΔV_0.5_ of 10.8 ± 2.0 mV (*n =* 3) was evident (Figure 5C,D).

### 2.4. Computational Modeling of the Brevetoxin–Nav Channel Complex

Alanine substitutions of 22 residues in helices IS6, IVS5, and IVS6 of the Nav1.2 channel resulted in a 2–3-fold change in the binding affinity of [42-^3^H]-PbTx-3, with small confidence intervals [12]. Using the cryo-EM structure of the hNav1.2 channel [27], we identified several key residues, including four in IS6 (M402, L407, F414, and Y415), two in IVS5 (G1664 and L1665), and one in IVS6 (Y1771), that face the lipid-exposed cleft between repeat domains I and IV. We utilized this information to initially position PbTx-2 in the cryo-EM structure. PbTx-2 and PbTx-3 share a similar structure, differing only by a single terminal CHO group in PbTx-2 and a CH_2_OH group in PbTx-3 (as depicted in Figure 1). This subtle difference is unlikely to affect their binding mode. Hence, we decided to dock PbTx-2, as its crystal structure is available in the Cambridge Structural Database [28] (CCDC 1106396; CSD code: BATLAJ).

The lengths of the PbTx-2 and PbTx-3 toxins are comparable to the thickness of the lipid bilayer. PbTx-3 has only one hydroxyl group located at the ladder top; this hydrophilic group may serve as a float when the toxin approaches the channel from the extracellular space. To account for this, we oriented PbTx-2 with its CHO group, which substitutes the CH_2_OH group in PbTx-3, at the extracellular part of cleft I/IV. As there are currently no experimental data on the channel’s interaction with individual groups of brevetoxins, we did not impose any specific distance constraints between PbTx-2 atoms and channel residues. Instead, we imposed constraints between the ligand and channel side chains (see Section 4).

**Figure 6:**
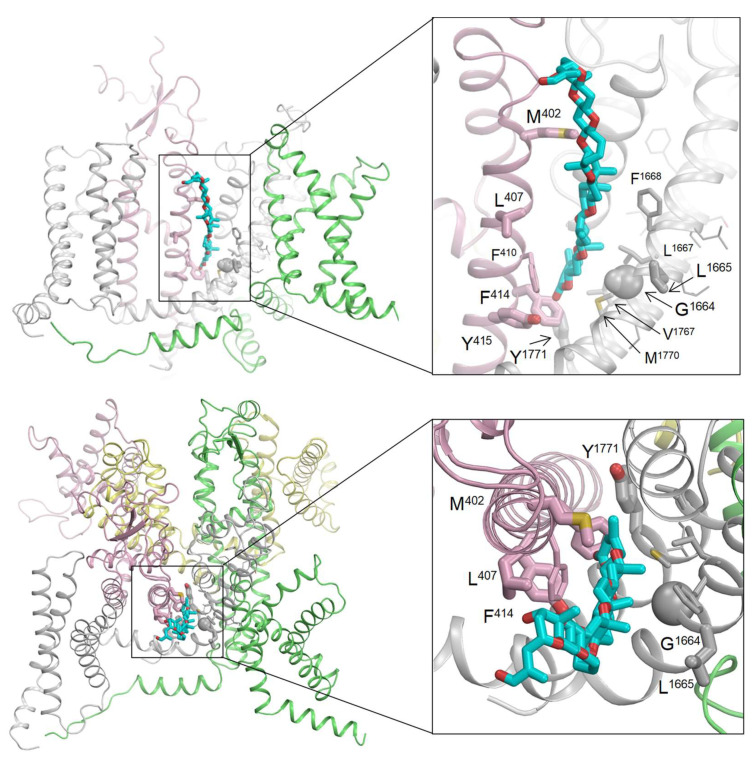
Membrane (top) and extracellular views of brevetoxin PbTx-2 docked into the cryo-EM structure of the Nav1.2 channel. The channel repeats I, II, III, and IV are pink, yellow, green, and gray, respectively. Brevetoxin is depicted with cyan carbons and red oxygens in stick form. Brevetoxin-sensing residues detected through ASM [12] are colored according to their respective backbones. Residues that are in direct contact with PbTx-2 are shown as sticks or spheres (Gly). Thick sticks represent residues whose alanine substitutions cause a significant impact on brevetoxin with small confidence intervals, whereas thin sticks represent residues whose replacement by alanine causes Kd changes with large confidence intervals. Residues that have a significant impact on brevetoxin binding with small confidence intervals but are not in direct contact with the ligand are shown as lines.

The toxin-bound channel has been modeled through intensive Monte Carlo energy minimizations, revealing that PbTx-2 fits seamlessly into the lipid-exposed cleft between repeat domains I and IV. This positioning enables direct contact with all seven brevetoxin-sensing residues in the cleft between domains I/IV, which are depicted as thick sticks or spheres in Figure 6. Additionally, several residues, including F410 in IS6, L1667 and F1668 in IVS5, and V1767 and M1770 in IVS6, occurred in direct contact with brevetoxin. Represented in Figure 6 as thin-width sticks, these residues demonstrate a small increase in Kd when substituted with alanine, but their confidence intervals are large.

The 15 remaining residues that are sensitive to brevetoxin are located within the pore module and do not directly interact with the toxins bound in the cleft. These residues, depicted as lines in Figure 6, are mainly involved in intersegment contacts. Replacing these residues with alanine could modify these contacts, potentially causing a shift in the balance between different channel states.

## 3. Discussion

We investigated the effects of PbTx-3 and brevenal on the function of recombinant voltage-gated sodium channel subtypes that are representative of central and peripheral neurons, as well as skeletal and cardiac muscle. These Nav channel subtypes were overexpressed in mammalian cell lines and functionally assessed using dialyzed whole-cell automated patch-clamp under quasi-physiological conditions. Additionally, we utilized computational modeling to study the interaction between PbTx-2 and Nav1.2 channels.

The functional effects of PbTx-3 and brevenal on macroscopic Nav currents can be summarized as follows: (1) enhancement of the late current component/Yinf; (2) inhibition of peak currents/Gmax; (3) changes to the voltage dependence of activation/V_0.5_ and delayed open channel inactivation/τ_inact_ (Table 2). Increasing the late current component, left-shifting V_0.5_, and/or delaying inactivation would concomitantly increase Nav channel activity, whereas peak current decreases and right-shifting V_0.5_ are broadly inhibitory to Nav activity. Although seemingly opposed, these features are commonplace and often overlap amongst many Nav channel agonists, including steroidal toxins and venom-derived peptides [29,30,31,32].

Within the Nav1 set analyzed here, Nav1.2 and Nav1.4 were most susceptible to PbTx-3 and brevenal modulation, whereas Nav1.5 and Nav1.7 appeared resistant. See Figure S1 for a side-by-side comparison.

### 3.1. Brevetoxin Modulation of Nav Currents

PbTx-3 causes Nav1.2 channels to mediate large late currents without apparent deleterious effects on its peak current amplitude. These effects are elicited at a potency that is congruent with the dissociation binding constants of PbTx-3 reported for rat brain synaptosomes [9] and Nav1.2 channels expressed in tsA201 cells [12]. In turn, PbTx-3 substantially inhibits Nav1.4’s peak currents, which, at saturating PbTx-3 concentrations, are superseded by marked enhancement of non-inactivating sustained currents (Figure 5A). The effects of PbTx-3 on macroscopic Nav1.4 currents are consistent with previous reports of T17 modification of crayfish and squid giant axons [33]. The open channel effects and hyperpolarizing shift in activation V_0.5_ led us to infer that the overall toxicity of PbTx-3 in skeletal muscle is due to its excitatory actions on Nav1.4. Thus, we surmise that brevetoxin’s excitotoxic actions may be underpinned by the enhancement of Nav1.2- and Nav1.4-mediated currents in the CNS and skeletal muscle, respectively.

The potencies obtained here for PbTx-3-induced I_peak_ inhibition or I_late_ enhancement of Nav1.5 channels are consistent with those observed in previous reports of single-channel analyses of rodent cardiomyocytes (at 30 μM) [34], and binding assays using photo-labeled PbTx-3 in heterologously expressed Nav1.5 channels [12]. Under our experimental conditions, Nav1.7 channels were most resistant to PbTx-3 and brevenal while still showing a hyperpolarizing shift in current activation in the presence of PbTx-3. This PNS Nav isoform underlies ~90% of the TTX-sensitive current in nodose ganglia vagal afferent neurons [34] in which single-channel recordings verified a similar leftward shift upon exposure to PbTx-3 (500 nM) [13]. At the single-channel level, a prominent effect of PbTx-3 modification of nodose Navs was the appearance of lower conductance (sub-conductances; 10.7 pS and 21.2 pS) levels that could be correlated with the PbTx-3-dependent peak current inhibition observed in our macroscopic recordings. Under our experimental conditions, whole-cell currents mediated by PbTx-3- and brevenal-treated recombinant Nav1.7 channels in CHO-K1 cells did not confer enhancement to the late current component nor overt changes to inactivation kinetics. In contrast, Jeglitsch et al. (1988) reported a ~2-fold increase in the mean apparent single-channel open time of nodose ganglion neuron Navs at all membrane potentials [13]. This observation could be attributed to the presence of other brevetoxin-sensitive Nav channel isoforms in these neurons [35,36] or potential variations in the auxiliary Nav subunit composition between native and recombinant systems. Hence, the results reported here and published previously are consistent with a lack of peripheral and/or cardiotoxic effects reported for the brevetoxins.

#### Brevetoxin Binding to Nav Channels

By incorporating published ASM [12], we were able to construct a computational model that reproduces the critical features of PbTx-2 binding to the lipid-exposed side of interface I/IV in Nav1.2.

The multiple-sequence alignment of the Nav1.2, Nav1.4, Nav1.5, and Nav1.7 channels reveals that out of the seven residues that directly interact with the toxin in our model, only one residue in Nav1.2 (L407) is substituted by valine in Nav1.4 (V429) and Nav1.7 (V384) channels. Thus, this observation alone cannot account for why the Nav1.5 channel is less sensitive to brevetoxin compared to Nav1.2 and Nav1.4 channels [12].

Among the brevetoxin-sensing residues, certain alanine substitutions would indirectly affect the toxin binding. Two Nav1.2 residues, S1758 and I1760, located in the extracellular half of helix IVS6, are substituted in Nav1.4 with C1579 and Y1581, respectively. Additionally, Nav1.2 residue S1758 is occupied in Nav1.5 by T1754. These findings suggest that the distinct brevetoxin sensitivity of the four channels studied here may be only partially explained by the differential chemical properties of the candidate brevetoxin-sensing residues identified through ASM analyses.

Differences in the gating properties of the different Nav isoforms influencing intersegment contacts between brevetoxin-sensing residues beyond cleft I/IV are thus more likely to underpin differing sensitivities extracted from functional analyses. It is worth noting that the available ASM analysis was primarily focused on helices IS6, IVS5, and IVS6, and partially on the extracellular loop IVS5-S6 (as shown in Figure 3 in [12]). These experiments identified several brevetoxin-sensing residues whose substitutions would allosterically affect toxin binding. However, other residues beyond cleft I/IV, which were not examined in the mutational analyses, may also impact brevetoxin action through allosteric effects.

### 3.2. Brevenal Modulation of Nav Currents

To our knowledge, this is the first detailed characterization of the modulation of Nav-mediated currents by brevenal. Contrary to PbTx-3’s agonistic effects on recombinant Nav currents, brevenal’s actions were largely inhibitory (i.e., decreasing I_peak_ and/or right-shifting V_0.5_, Table 2) and occurred at substantially higher concentrations (Figure 3 and Figure S1). This is apparent even in the case of Nav1.4, for which enhancement of I_late_ was observed. However, in contrast to PbTx-3, brevenal exposure consistently induced right shifts in V_0.5_, whether extra- or intracellularly applied. Furthermore, brevenal’s inhibitory actions on recombinant Nav channels are consonant with previous reports of native sodium channel blocking [19].

With regard to marked differential potency compared to PbTx-3, higher concentrations of the shorter polyether brevenal were required to elicit appreciable Nav channel modulation, which is consistent with previous studies demonstrating that longer-chain brevetoxin analogs bind Nav channels more potently than shorter ones [18].

### 3.3. Brevenal Antagonizes Brevetoxins

Amongst the marine LFPs, both brevetoxins and ciguatoxins are Nav channel activators, whereas gambierol acts as a Kv channel inhibitor. The lipophilic polyether Kv channel blocker gambierol (*Gambierdiscus Toxicus*) is structurally related to breve- and cigua- toxins but does not modulate Nav-mediated currents [37,38]. In native cells, competitive binding experiments support breve- and cigua-toxins binding to Nav channel site 5 [39]. Similar to brevenal, gambierol acts as a competitive antagonist to both breve- and cigua- toxin actions to Nav channels, consistent with overlapping binding to site 5 and/or potential negative allosteric actions to Nav channels [40].

We show that the addition of brevenal to the intracellular solution results in Nav1-mediated currents becoming resistant to PbTx-3 modulation. Earlier studies have demonstrated that brevenal reduces brevetoxin binding in radioligand assays [22,23]. Since mutational analyses to determine brevenal’s binding site have not been performed, we did not attempt to dock this toxin to Nav1.2. Nevertheless, in light of the Nav1.2/PbTx-2 computational model presented here, our functional findings provide a plausible mechanism for the anti-brevetoxins effects reported previously, whereby brevenal competes with PbTx for binding to the lipid-exposed side of Nav1 interface I/IV. This further suggests their potential use as regulators of organ and tissue metabolism, serving as both positive and negative regulators [2].

Given that brevenal is a natural product under investigation as a therapy for chronic respiratory diseases, such as cystic fibrosis or asthma, future mutagenesis studies to elucidate its precise binding site and anti-toxin mechanisms are warranted.

## 4. Materials and Methods

### 4.1. Materials

All salts were purchased from Sigma.

Brevetoxin PbTx-3 (CAS 85079-48-7, Calbiochem) was purchased from Millipore (Merck KGaA, Darmstadt, Germany), and reconstituted as a 10 mM stock solution in ethanol. Brevenal was provided by MARBIONC University of North Carolina, Wilmington. Brevenal samples were provided with accompanying NMR and high-resolution mass spectrometry analyses to assure proof of purity and composition. PbTx-3 and brevenal were diluted in external and/or internal solution to the desired concentration.

Recording solutions: The extracellular solution contained (in mM) 140 NaCl, 5 KCl, 2 CaCl_2_, 2 MgCl_2_, 10 glucose, and 10 HEPES (pH 7.4 with NaOH, 298 ± 3 mOsmol/k g). Control intracellular solution (in mM): 60 CsF, 70 CsCl, 10 EGTA, 10 glucose, and 10 HEPES (pH 7.2 with CsOH, 285 ± 3 mOsmol/kg). Intracellular exposure to brevenal (1 μM) was achieved by including it in the control intracellular solution during chip filling.

HEK293 cells heterologously expressing human Nav1.2 (Scottish Biomedical, a kind gift from the Petrous lab, Florey Institute of Neuroscience and Mental Health, Melbourne, VIC Australia) and Nav1.5 (ChanTest Corp., Cleveland, OH, USA) were cultured in DMEM containing 10% *v*/*v* FBS and selection antibiotics to enable stable expression as per the manufacturer’s recommendation. CHOK1 expressing Nav1.4 and Nav1.7 (ChanTest) was cultured in DMEM/F12 containing 10% v/v FBS and selection antibiotics supporting stable expression as recommended by the manufacturer. Cells were maintained in a humidified 5% CO_2_ incubator at 37 °C, grown in T25 flasks to 70–80% confluence, and passaged every 3–4 days using TrypLE Express (Invitrogen). After harvesting with TrypLE, cells were resuspended to 10^5^ cells/mL in cold extracellular solution and allowed to recover for 30 min at 4 °C before recording.

### 4.2. Methods

Electrophysiology: Automated patch-clamp (APC) recordings were performed in a PatchLiner Octo (Nanion Technologies GmbH, Munich, Germany) equipped with two EPC-10 quadro patch-clamp amplifiers (HEKA Electronik, Lambrecht/Pfalz, Germany). PatchControl HT (Nanion) was used for cell capture, seal formation, and establishment of the whole-cell configuration, whilst voltage was controlled and currents were sampled with PatchMaster (HEKA Electronics). Recordings were performed under the whole-cell configuration using single-hole planar medium resistance NPC-16 chips (R_chip_, resistance of ~2.5 MΩ) at room temperature (22–24 °C).

Cells between passages 6 and 12 were used. Recordings where seal resistance (R_seal_) was >500 MΩ and access resistance were <3 × R_chip_ were considered acceptable. Chip and whole-cell capacitance were fully compensated, and series resistance (Rs) compensation (70%) was applied via the Auto Rs Comp function. Recordings were acquired with PatchMaster (HEKA Elektronik) and stored on a computer running PatchControl HT software (Nanion Technologies GmbH).

Nav currents were evoked by a 25 ms test pulse to −20 mV (Vh = −120 mV; 0.1 Hz). Extracellular drug applications were performed by exchanging the whole bath with the test compounds diluted in extracellular solution to the required concentration during constant monitoring (25 ms, −20 mV, Vh −120 mV, 0.1 Hz). The control solution included 0.1% ethanol to account for non-specific vehicle effects. For concentration–response curves (CRCs), compounds at incrementally higher concentrations were applied to the extracellular bath solution and allowed to equilibrate for 5 min. The average current amplitude from the last five test pulses at each concentration was used for analysis.

### 4.3. Data Analysis

APC data were analyzed using PatchMaster (Nanion) and Igor (WaveMetrics, Portland, OR, USA) software. Nav peak and late currents measured in the presence of increasing amounts of PbTx-3 and brevenal were divided by the current in control conditions (ICtr) to generate CRCs. CRCs were fitted with a Hill equation of the form:

IComp/ICtr = Ymin + *m*/(1 + (EC_50_/[Comp])^n^)

where EC_50_ is the half-maximal inhibitory (IC_50_) or effective (EC_50_) concentration, the hill coefficient (*nH*) was set to 0.5, and *m* is the maximal effect attained.

The inactivation time constant τ_inact_, and the horizontal asymptote Y_inf_, corresponding to the amplitude of the late current component, were estimated by exponential fits of the form I = Y_inf_ + A exp [−(t − t_0_)/τ_inact_], where A is the maximal current amplitude.

Peak I–V curves were fitted using:

I_(V)_ = (V − V_rev_) × Gmax/(1 + exp ((V_0.5_ − V)/V_slope_)

where I is the macroscopic current, V is the command potential, V_rev_ is the reversal potential (mV), Gmax is the maximal conductance, V_0.5_ is the half-activation potential (mV), and V_slope_ is the slope factor (mV/e-fold).

All summary data are presented as mean ± SEM (n), where n is the number of independent determinations obtained from at least two different cell passages per group. Data sets underwent Shapiro–Wilk normality (when *n*  ≥  5) to verify if samples were normally distributed and to designate downstream statistical analyses as parametric or nonparametric. All unpaired datasets were deemed parametric, and therefore, comparison between two groups was performed by Student’s t-test, while one-way ANOVA was used to compare across >3 groups. In the latter test, F value significance (*p*  <  0.05) and variance homogeneity (*p* > 0.05) supported the implementation of post hoc Dunnett’s and Tukey’s test for multiple group comparisons.

### 4.4. Computational Modeling

We used the cryo-EM structure of the Nav1.2 channel [27] (PDB ID: 6J8E) to dock PbTx-2 in the lipid-exposed cleft between repeat domains I and IV. The ZMM program (www.zmmsoft.ca (accessed on 15 January 2023)).was employed to dock the toxin. The energy was calculated using the AMBER force field [41,42] with distance- and environment-dependent dielectric function [43]. The toxin–channel complex was optimized with Monte Carlo energy minimizations [44]. Atomic charges in PbTx-2 were calculated with the MOPAC program [45].

Ligand-side chain constraints were implemented to address the lack of experimental data on specific contacts between PbTx-2 and Nav1.2. These constraints were designed to bias the proximity of PbTx-2 to four channel residues located in the I/IV cleft, which are known to have a significant impact on toxin binding when substituted with alanine [12]. Each constraint specified a channel residue (M402, F414, G1664, or Y1771) and set an upper distance limit of 5 Å between the channel sidechain and PbTx-2. At the beginning of each round of energy minimization, the ZMM program identified the closest pairs of atoms between PbTx-2 and the side chains. It is important to note that these atom pairs may have been automatically changed during the computational process. To maintain the structural integrity of the channel backbones in relation to the cryo-EM structure, we applied “pin” constraints. These constraints utilize a flat-bottom parabolic energy function, permitting an α-carbon to deviate by up to 1 Å from its experimental position without any penalties. Any deviations beyond this limit result in an energy penalty.

The sampling protocol involved randomizing the torsion angles of the channel side chains, as well as the position and orientation of the toxin and the torsion angles of substitutions in the toxin’s “tail” ring (Figure 1). All generalized coordinates, including the channel backbone torsions and PbTx-2’s torsional and bond angles, were flexible during energy minimizations. Monte Carlo energy minimizations were stopped when the energy did not improve in the last 1000 consecutive minimizations. Additional computational details can be found elsewhere [46,47].

## 5. Conclusions

Complementary binding studies in native tissues and functional studies in recombinant systems are crucial in establishing a link between the structure of proteins and their physiological functions. These studies help in understanding how the protein structure affects its function in living organisms. In the case of brevetoxins and brevenal and Nav isoforms, the differential functional effects observed are probably due to the intrinsic functional differences between the isoforms, rather than differences in pharmacophores. Thus, a combination of these two types of studies provides valuable insights into the mechanisms underlying protein function in vivo.

Our results lay the groundwork for future mutational analyses, aiming to develop comprehensive models of sodium channels. These models will cover natural, derivatized, and synthetic brevetoxins and brevenal. Through our investigation of the binding and function of brevetoxin and brevenal on recombinant Nav channel isoforms, we have gained valuable insights. These insights are crucial for identifying the specific organs and tissues susceptible to toxicity, as well as potential antagonists. Additionally, they deepen our understanding of the various illnesses caused by brevetoxins. Considering these findings, a detailed therapeutic matrix can be envisioned, providing a comprehensive framework for therapeutic interventions and treatment strategies.

The current study provides a detailed characterization of the effects of PbTx-3 on Nav channel kinetics, shedding light on the mechanisms underlying Nav channel gating and their impact on excitability. The complexity of Nav channel function and regulation is underscored, as different LFPs affect Nav channel isoforms in distinct ways. The molecular docking used offers valuable insights into the molecular mechanisms involved in the functional modulation of Nav channels by brevetoxins, which could guide future drug development efforts. Importantly, these findings have significant implications for the development of novel Nav channel modulators with potential therapeutic applications. Together, these results advance our understanding of Nav channel physiology and pave the way for further investigations into the intricate regulation of these channels.

## Figures and Tables

**Table 1:** PbTx-3 and brevenal modification potency (maximal fold change, FC^Max^) of peak and late Nav-mediated currents.

		PbTx-3			Brevenal		
			FC^Max^	EC_50_ (M)		FC^Max^	EC_50_ (M)
Nav1.2	I_peak_	∅	-	-	↓	0.6 ± 0.1 *	1.8 ± 0.7 × 10^−8^
	I_late_	↑	3.0 ± 0.1 *	5.3 ± 1.8 × 10^−12^	∅	-	-
Nav1.4	I_peak_	↓	0.03 ± 0.03 ‡	9.1 ± 2.7 × 10^−10^	↓	0.1 ± 0.1 ‡	7.7 ± 2.2 × 10^−9^
	I_late_	↑	4.4 ± 0.2 †	4.1 ± 1.0 × 10^−9^	↑	4.1 ± 0.8 †	1.0 ± 0.3 × 10^−7^
Nav1.5	I_peak_	↓	0.2 ± 0.1 *	2.2 ± 0.7 × 10^−9^	↓	0.4 ± 0.1 ‡	1.7 ± 0.5 × 10^−8^
	I_late_	∅	1.8 ± 0.1	-	∅	1.3 ± 0.2	-
Nav1.7	I_peak_	↓	0.4 ± 0.1 †	1.6 ± 0.9 × 10^−10^	∅	-	-
	I_late_	∅	0.6 ± 0.1		∅	-	-

∅ no change, ↑ increased, ↓ decreased. Data represent mean ± SEM, *n* = 5 for all determinations. One-way ANOVA determined significance with Dunnett’s multiple comparisons test. Control vs. FC^Max^: * *p* < 0.05; † *p*< 0.005; ‡ *p* < 0.0001.

**Table 2:** Summary of PbTx-3 and brevenal modification of Nav-mediated currents.

	PbTx-3_o_						Brevenal_o_					
	I_peak_	G_max_	I_late_	Y_inf_	τ_inact_	V_0.5_	I_peak_	G_max_	I_late_	Y_inf_	τ_inact_	V_0.5_
Nav1.2			↑	↑			↓	↓				→
Nav1.4	↓	↓	↑	↑	↑	←	↓	↓	↑	↑	↑	→
Nav1.5	↓	↓				→	↓	↓				
Nav1.7	↓	↓				←						←

↑ increase, ↓ decrease, ← left shift, → right shift.

## Data Availability

All study data are included in the article and/or SI Appendix.

## Supplementary Materials

The following supporting information can be downloaded at: https://www.mdpi.com/article/10.3390/md21070396/s1. Table S1: Neuronal Nav current inactivation parameters determined in the absence and presence of PbTx-3 and brevenal; Table S2: Skeletal muscle Nav current inactivation parameters determined in the absence and presence of PbTx-3 and brevenal; Table S3: Cardiac muscle Nav current inactivation parameters determined in the absence and presence of PbTx-3 and brevenal; Figure S1: PbTx-3 and brevenal potency comparison between human Nav channel isoforms.
