# Supplementary material for: Brevetoxin versus Brevenal Modulation of Human Nav1 Channels
Source: Mar Drugs. 2023 Jul 7;21(7):396. doi: 10.3390/md21070396 (PMC10382042; doi:10.3390/md21070396)
Supplement: Supplementary file 1 [file marinedrugs-21-00396-s001.zip › marinedrugs-2442359-supplementary.pdf]

marine drugs

Supplementary Materials

Brevetoxin versus Brevenal Modulation of Human Nav1 Channels

Rocio K. Finol-Urdaneta [1,*], Boris S. Zhorov [2,3,4], Daniel G. Baden [5] and David J. Adams [1,*]

[1] Illawarra Health & Medical Research Institute (IHMRI), Faculty of Science, Medicine and Health, University of Wollongong, Wollongong, NSW 2522 Australia;
[2] McMaster University, Hamilton, Ontario, Canada;
[3] I.M. Sechenov Institute of Evolutionary Physiology and Biochemistry, Russian Academy of Sciences, Saint Petersburg.
[4] Shemyakin-Ovchinnikov Institute of Bioorganic Chemistry Russian Academy of Sciences, Moscow
[5] University of North Carolina Wilmington MARBIONC, North Carolina USA;
* Correspondence: RKF-U rfinolu@uow.edu.au; DJA djadams@uow.edu.au

Abstract: Brevetoxins (PbTx) and brevenal are marine ladder-frame polyethers (LFPs). PbTx binds to and activates voltage-gated sodium (Nav) channels in native tissues, whereas brevenal antagonizes these actions. However, the effects of PbTx and brevenal on recombinant Nav channel function have not been systematically analyzed. In this study, the PbTx-3 and brevenal modulation of tissue-representative Nav channel subtypes Nav1.2, Nav1.4, Nav1.5, and Nav1.7 was examined using automated patch-clamp. While PbTx-3 and breveval elicit concentration-dependent and subtype-specific modulatory effects, PbTx-3 is >1000-fold more potent than brevenal. Consistent with effects observed in native tissues, Nav1.2 and Nav1.4 channels were PbTx-3- and brevenal-sensitive, whereas Nav1.5 and Nav1.7 appeared resistant. Interestingly, incorporation of brevenal in the intracellular solution caused Nav channels to become less sensitive to PbTx-3 actions. Furthermore, we generated a computational model of PbTx-2 bound to the lipid-exposed side of the interface between domains I and IV of Nav1.2. Our results are consistent with competitive antagonism between brevetoxins and brevenal, setting a basis for future mutational analyses of Nav channels' interaction with brevetoxins and brevenal. Our findings provide valuable insights into the functional modulation of Nav channels' by brevetoxins and brevenal, which may have implications for the development of new Nav channel modulators with potential therapeutic applications.

Keywords: Brevenal; Brevetoxins; Nav; Ladder-frame polyethers; PbTx-3; PbTx-2; Voltage-gated sodium channels

Citation: To be added by editorial staff during production.

Academic Editor: Firstname Last-name

Received: date
Revised: date
Accepted: date
Published: date

Copyright: © 2023 by the authors. Submitted for possible open access publication under the terms and conditions of the Creative Commons Attribution (CC BY) license (https://creativecommons.org/licenses/by/4.0/).

Supplementary Materials: The following supporting information can be downloaded at: https://www.mdpi.com/xxx/s1. Table S1. Neuronal Nav current inactivation parameters determined in the absence and presence of PbTx-3 and brevenal; Table S2. Skeletal muscle Nav current inactivation parameters determined in the absence and presence of PbTx-3 and brevenal; Table S3. Cardiac muscle Nav current inactivation parameters determined in the absence and presence of PbTx-3 and brevenal; Figure S1. PbTx-3 and brevenal potency comparison between human Nav channel isoforms.

Table S1. Neuronal Nav current inactivation parameters determined in the absence and presence of PbTx-3 and brevenal.

		**PbTx-3**				**Brevenal**			
	(M)	0	10^{-12}	10^{-9}	10^{-6}	0	10^{-9}	10^{-6}	10^{-5}
Nav1.2	y_{inf}	0.06±0.01	0.13±0.01*	0.19±0.02*	0.28±0.4*	0.09±0.03	0.08±0.04	0.10±0.07	0.12±0.06
	τ_{inact} (ms)	0.80±0.07	0.88±0.1	0.76±0.05	0.64±0.14	0.71±0.07	0.74±0.8	0.65±0.08	0.75±0.10
Nav1.7	y_{inf}	0.02±0.01	0.03±0.01	0.03±0.01	0.04±0.01	0.06±0.01	0.07±0.01	0.07±0.01	0.07±0.01
	τ_{inact} (ms)	0.64±0.06	0.60±0.05	0.63±0.04	0.66±0.11	0.81±0.06	0.76±0.05	0.85±0.06	0.93±0.09

Inactivation parameters were extracted from single exponential fits to the current decay.

One-way ANOVA of compound vs Ctr: * $p < 0.05$; ** $p < 0.005$; Data represent mean ± SEM, n = 4 for all determinations.

Table S2. Skeletal muscle Nav current inactivation parameters determined in the absence and presence of PbTx-3 and brevenal.

		**PbTx-3**				**Brevenal**			
	(M)	0	10^{-12}	10^{-9}	10^{-6}	0	10^{-9}	10^{-6}	10^{-5}
Nav1.4	y_{inf}	0.04±0.01	0.07±0.01	0.16±0.02*	0.20±0.02*	0.03±0.01	0.09±0.01	0.14±0.03*	0.19±0.03*
	τ_{inact} (ms)	0.60±0.04	0.56±0.05	0.61±0.04	1.37±0.05**	0.68±0.1	0.71±0.11	0.77±0.0.08	1.16±0.15**

Inactivation parameters were extracted from single exponential fits to the current decay.

One-way ANOVA of compound vs Ctr: * $p < 0.05$; ** $p < 0.005$; Data represent mean ± SEM, n = 4 for all determinations.

Table S3. Cardiac muscle Nav current inactivation parameters determined in the absence and presence of PbTx-3 and brevenal.

		**PbTx-3**				**Brevenal**			
	(M)	0	10^{-11}	10^{-9}	10^{-7}	0	10^{-9}	10^{-6}	10^{-5}
Nav1.5	y_{inf}	0.09±0.01	0.20±0.03	0.29±0.03**	0.45±0.05*	0.03±0.01	0.03±0.01	0.03±0.01	0.02±0.01
	$A1_{Rel}$	0.89±0.01	0.82±0.05	0.72±0.01**	0.71±0.06	0.71±0.07	0.73±0.07	0.77±0.07	0.77±0.05
	$\tau 1_{inact}$ (ms)	0.45±0.01	0.46±0.03	0.48±0.06	0.45±0.02	0.46±0.01	0.45±0.01	0.46±0.01	0.44±0.01
	$A2_{Rel}$	0.11±0.01	0.18±0.05	0.28±0.01**	0.29±0.06	0.03±0.01	0.03±0.01	0.03±0.01	0.02±0.01
	$\tau 2_{inact}$ (ms)	4.35±0.49	2.42±0.52	2.67±0.78	1.48±0.31	2.05±0.16	1.77±0.34	2.05±0.21	1.79±0.06

Inactivation parameters were extracted from single exponential fits to the current decay.

One-way ANOVA of compound vs Ctr: * $p < 0.05$; ** $p < 0.005$; Data represent mean ± SEM, n = 4 for all determinations.

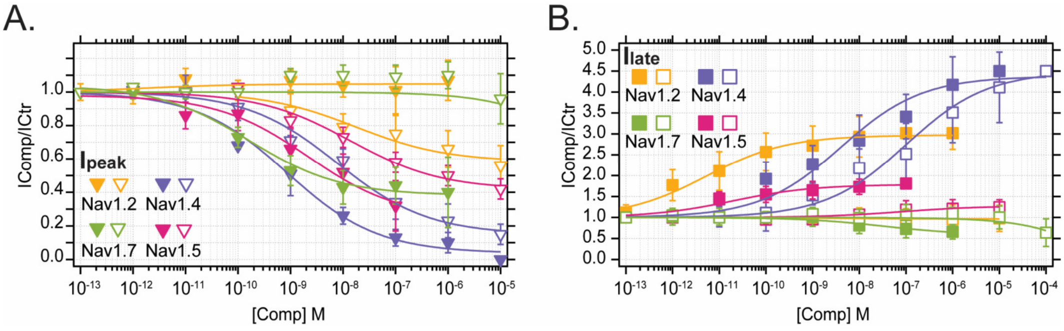

Figure S1. PbTx-3 and brevenal potency comparison between human Nav channel isoforms. Solid symbols PbTx-3, open symbols brevenal.
